# A Review of Molecular Mechanisms of the Anti-Leukemic Effects of Phenolic Compounds in Honey

**DOI:** 10.3390/ijms131115054

**Published:** 2012-11-15

**Authors:** Murtala B. Abubakar, Wan Zaidah Abdullah, Siti Amrah Sulaiman, Ang Boon Suen

**Affiliations:** 1 Department of Physiology, School of Medical Sciences, Universiti Sains Malaysia, 16150 Kubang Kerian, Kelantan, Malaysia; E-Mail: bsang@kb.usm.my; 2 Department of Haematology, School of Medical Sciences, Universiti Sains Malaysia, 16150 Kubang Kerian, Kelantan, Malaysia; E-Mail: wzaidah@kb.usm.my; 3 Department of Pharmacology, School of Medical Sciences, Universiti Sains Malaysia, 16150 Kubang Kerian, Kelantan, Malaysia; E-Mail: sbsamrah@kb.usm.my

**Keywords:** leukemia, honey, phenolic compounds, cell cycle, apoptosis, cell growth and proliferation

## Abstract

Hematologic malignancies constitute about 9% of all new cases of cancers as reported via the GLOBOCAN series by International Agency for Research on Cancer (IARC) in 2008. So far, the conventional therapeutic and surgical approaches to cancer therapy have not been able to curtail the rising incidence of cancers, including hematological malignancies, worldwide. The last decade has witnessed great research interest in biological activities of phenolic compounds that include anticancer, anti-oxidation and anti-inflammation, among other things. A large number of anticancer agents combat cancer through cell cycle arrest, induction of apoptosis and differentiation, as well as through inhibition of cell growth and proliferation, or a combination of two or more of these mechanisms. Various phenolic compounds from different sources have been reported to be promising anticancer agents by acting through one of these mechanisms. Honey, which has a long history of human consumption both for medicinal and nutritional uses, contains a variety of phenolic compounds such as flavonoids, phenolic acids, coumarins and tannins. This paper presents a review on the molecular mechanisms of the anti-leukemic activity of various phenolic compounds on cell cycle, cell growth and proliferation and apoptosis, and it advocates that more studies should be conducted to determine the potential role of honey in both chemoprevention and chemotherapy in leukemia.

## 1. Background

The International Agency for Research on Cancer (IARC) via its GLOBOCAN series estimated that about 12.7 million people were diagnosed with cancer and approximately 7.6 million died from the disease in 2008 [1]. Hematologic malignancies, consisting of leukemia, Hodgkin lymphoma, non-Hodgkin lymphoma and myeloma, account for about 9% of all cancers and are the fourth most commonly diagnosed cancers in both men (after prostate, lung and colorectum) and women (after breast, lung, and colorectum) in economically advanced nations of the world [2]. Cancer chemoprevention refers to prevention of cancer using a number of intervention strategies such as administration of either a pharmacologic agent or a chemical constituent of natural diet, the ultimate goal of which is to prevent, halt or reverse the development of cancer (carcinogenesis) at different levels [3,4]. Phenolic compounds (polyphenols) are made up of various groups of metabolites commonly found in the human diet and represent different forms of chemo-preventive agents that have been extensively studied in the last decade [3]. They are widely distributed in the plants and found naturally in a variety of fruits and vegetables [5] as well as in honey, propolis and royal jelly [6–8]. The last few years have witnessed a tremendous research interest in phenolic compounds [3], this is because there are available evidences from *in vitro in vivo*, and epidemiological studies as well as from clinical trials that are supporting their role as antioxidants, anti-inflammatory, and anti-carcinogenic agents as well as in the treatment of heart diseases [9–17]. It is now fully established that consumption of an appropriate diet can prevent development of cancer [3,18]. So far, the conventional therapeutic and surgical modalities of treatment of cancers have failed to check the increased incidence of most cancers; it is therefore, as a matter of urgency, necessary to establish sound action plans for the purpose of reducing or preventing the rampant incidence of cancers. Use of chemo preventive agents such as polyphenols may be one such strategy. Polyphenols have met the above definition of chemoprevention by virtue of their ability to inhibit the different stages of cancer development *i.e.*, tumor initiation, promotion and progression, which could be achieved through inactivation of carcinogen or inhibition of its formation, growth inhibition, cell cycle arrest, induction of apoptosis and differentiation, angiogenesis inhibition, and anti-oxidation or combination of these effects [3].

### 1.1. Cell Cycle

**Figure 1:**
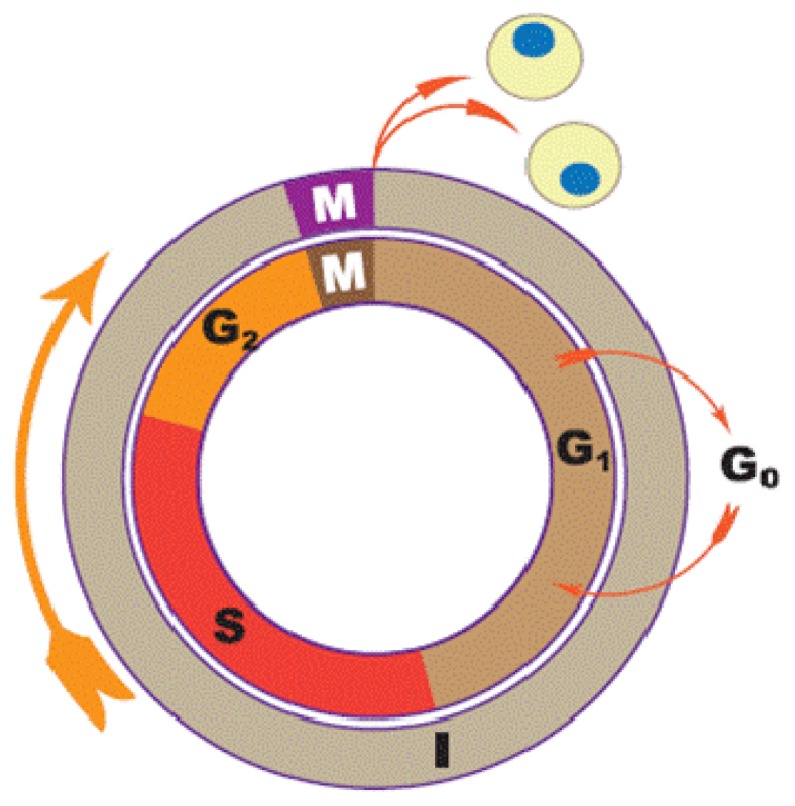
Four phases of cell cycle (G_1_-S-G_2_-M).

**Figure 2:**
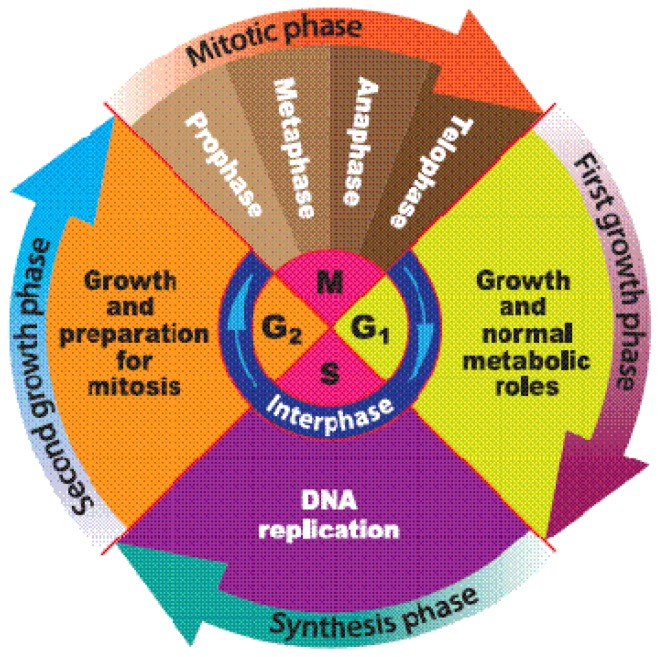
Relationship between the different phases of cell cycle.

The cell cycle constitutes a series of molecular events of cell division during which cells grow and divide to give rise to new daughter cells. It is generally divided in to four phases, namely G_1_ (gap 1 phase), S (synthesis phase), G_2_ (gap 2 phase), and M (mitosis phase). G_1_ and G_2_ are two breaks with variable duration separating the S and M phases as depicted in Figures 1 and 2; this gives the cycle its general illustration of G_1_-S-G_2_-M [19,20]. Disruption of execution of normal cell cycle plays a vital role in the development of cancer [21,22]. The cell cycle is regulated by two vital classes of molecules: The cyclin (cyclin A–cyclin T) and the cyclin-dependent protein kinases (CDK 1–CDK 9). CDK/cyclin complexes are in turn excited by CDK-activating kinase (CAK) also known as cdk7/cyclin H. Growth of cell is necessary before S phase of the cell cycle begins and c-myc in addition to its role in both cell cycle and cell proliferation, is believed to play a key role in this important event of increase in cell volume [19,23,24]. G_o_ phase is a resting phase during which cells stop undergoing cell cycle including replication. These nonreplicating cells are either quiescent or senescent cells. It is now believed that anticancer agents that target cell cycle may significantly determine the success of cancer chemotherapy, as well as provide clues in finding a complete cure for many tumors, hence, targeting the different stages of cell cycle could play a vital role in the discovery of new anticancer drugs [18,19,21].

### 1.2. Apoptosis

Apoptosis or programmed cell death is a unique type of cell death observed in both physiological and pathological conditions. The hallmark features of apoptosis include both morphological and biochemical changes in the form of cell shrinkage, DNA fragmentation, membrane blebbings, chromatin condensation and loss of adhesion and rounding [25,26]. Apoptosis is regulated by various proteins, notably p53, IAPs, and caspase and Bcl families. It is believed that receptor and mitochondrial mechanisms of apoptosis are triggered in response to cancer treatment. Caspases that regulate apoptosis are classified in to initiator and effector caspases. Bcl-2 family proteins predominantly found in mitochondrial membrane play a vital role in mitochondria mediated apoptotic cell death and are made up of both anti-apoptotic (Bcl-2, Bcl-X_L_, and Mcl-1) and pro-apoptotic (Bax, Bck, and Bad) molecules as well as BH3 domain only (Bid, Bim, Puma and Noxa) molecules which are also believed to be pro-apoptotic proteins. IAPs comprise of molecules that exert inhibitory effects on activity of caspase-3, 7 and 9 [27].

The p53 gene is very vital in the regulation of both cell cycle and apoptosis; therefore, whenever there are insults to the cells in the form of growth factor deprivation, DNA damage or expression of oncogenes, it will be stimulated and result in either cell cycle arrest or apoptotic cell death [28]. The cell death induced by p53 upon its activation is believed to be mediated by the release of mitochondrial cytochrome c and Smac/DIABLO. The released cytochrome c appropriates the formation of caspase 9 and adapter protein Apaf-1. The activated caspase 9 in turn activates effector caspases-3 and -7 which eventually cause the cell death. The activation of caspases is aided by the regulatory effects of apoptogenic factors Smac/DIABLO on IAPs. The release of cytochrome c and Smac/DIABLO is mediated by the Bcl-2 family [27,29]. Another important molecular event is the inhibition of CDKs by two families of proteins namely: INK4 family consisting of p16^INK4a^, p15^INK4b^, p18^INK4c^ and p19^INK4d^ which specifically inhibits CDK4 and CDK6, and the CIP/KIP family which has stimulatory effect on p21^cip1/waf1^, p27^kip1^ and p57^kip2^[21]. p21^cip1^ is a well-known inhibitor of CDKs [30].

Apoptosis yields numerous hints concerning efficient cancer chemotherapy, and many of the reported anticancer agents exert their chemotherapeutic effects by way of inducing apoptotic cell death [25].

### 1.3. Honey

Honey is a natural sweet substance, produced by honey bees from the nectar of plants or from secretion of living parts of plants or excretion of plant-sucking insects on the living parts of plants, which the bees collect, transform by combining with specific substances of their own, deposit, dehydrate, store and leave in honeycombs to ripen and mature [31]. It has a long history of human consumption and is commonly used for both nutritional and medicinal purposes. Honey contains carbohydrates, protein and amino acids, vitamins, and phenolic compounds [32–38]. Although the anticancer effects of crude honey in various cancer types has been reported [39–44], to the author’s knowledge, no similar report is available on the effect of crude honey on leukemia and other hematologic malignancies. We present a review on the evaluation of biological activities of the various phenolic compounds in honey on cell cycle, cell growth and proliferation, as well as induction of apoptosis, with a view that subsequent elucidation of the molecular mechanisms of their actions will provide considerable hint for the development of newer cancer chemotherapeutic and chemo preventive agents. However, this report is not aimed at providing an exhaustive review on this subject matter as the presence of a number of polyphenols in honey is yet to be confirmed, it therefore only concentrates on those phenolic compounds already reported to be found in honey.

### 1.4. Major Phenolic Compounds Present in Honey

A bulk of the phenolic compounds found in honey is in the form of flavonoids [8]. However, other classes of phenolic compounds are also present in appreciable amount. Table 1 summarizes the different classes of phenolic compounds present in honey [3,7,45–49].

## 2. Molecular Mechanisms of Anti-Leukemic Activities of Individual Polyphenols in Honey on Different Cell Lines

Various leukemia cell lines have been reported as used in leukemia *in vitro* studies. Commonest among them include HL-60, K562, U937, NALM-6, MOLT-4 and recently discovered YCUB series. HL-60 cells, derived from a patient with acute promyelocytic leukemia, are human myelogenous leukemia cell lines with a phenotypic resemblance to their lineage *i.e.*, myeloblastic and promyelocytic cells. They are ideal for studying pharmacological agent or natural product-cell interactions and have characteristic apoptotic response to chemotherapeutic agents [50–53]. K562 cells are myeloid leukemia cell lines derived from a patient with chronic myeloid leukemia. They characteristically carry the Philadelphia chromosome, a cellular marker for chronic myeloid leukemia; this makes them ideal representatives of chronic myeloid leukemia cell lines [54]. U937 cells, phenotypically resembling their blast cells of their lineage were derived from a patient with diffuse histiocytic lymphoma and are representatives of monocyte-related leukemia/lymphoma [55]. NALM-6 cells were isolated from a patient with non-T, non-B acute lymphoblastic leukemia and are considered to be phenotypically pre-B cell lines [56]. MOLT-4 represent human leukemic cell lines established from a patient with T-lymphocytic leukemia relapsed after multidrug chemotherapy [57]. YCUB series is a collection of acute lymphoblastic leukemia (ALL) cell lines derived from pediatric patients with B precursor ALL. Popular among these cell lines include YCUB-2, YCUB-4, YCUB-5, and YCUB-8. YCUB-2 has t (17;19) and carries E2A-HLA fusion protein. YCUB-4 carries CD5, CD19, CD20, and HLA-DR positive surface markers. YCUB-5 (positive for CD10, CD13, CD19, and HLA-DR) has the abnormal karyotype with +*x*, +21. YCUB-8 established from a 13-year-old female has t (1;19) and is a relatively newly developed cell line [58,59] We therefore present anti-leukemic actions of individual polyphenols in each of these cell lines.

### 2.1. Quercetin

The role of quercetin (3,3′,4′,5,7-penta-hydroxyflavone) as an anti-cancer agent has been studied by Kang and Liang. They demonstrated a dose dependent inhibition of HL-60 cells by quercetin at a dose of between 10 and 80 μM. Following incubation with 10 μM Quercetin, the HL-60 cells displayed percentage growth inhibition of 17.1, 27.3, 40.1, and 52.7% after 24, 48, 72, and 96 h of treatment respectively. Analysis of the cell cycle revealed increase in number of cells in G_2_/M from 7.6% to 12.4, 19.1, and 23.5% after treatment with 20, 40 and 60 μM quercetin respectively, and a corresponding decrease in number of cells in G_o_/G_1_ cells from 46.2% to 40.2, 32.1, and 34.5% respectively, however, no significant effect was observed on the percentage count in S phase of the cycle after 24 h treatment. Moreover, they found a significant inhibition of the activities of membrane tyrosine protein kinase (TPK) and cytosolic protein kinase C (PKC) in HL-60 cells *in vitro*. On this basis, they concluded that the antiproliferative effect of quercetin may have to do with its inhibitory action on membrane TPK and/or cytosolic PKC [60]. Csokay and colleagues further elaborated on the molecular bases of antiproliferative action of quercetin. They demonstrated that treatment of K562 human leukemia cells with quercetin induced both apoptosis and differentiation as evident by internucleosomal DNA fragmentation. These effects were also dose and time-dependent with a dose of 55 μM quercetin capable of inducing DNA fragmentation after 1 h treatment and a dose of 550 μM quercetin capable of causing 45% of internucleosomal DNA fragmentation after prolonged treatment of up to 24 h duration. They ascribed these effects to two changes namely; reduction in inositol 1,4,5-triphosphate (IP_3_) concentration and down-regulation of expression of c-myc and ki-ras oncogenes in K562 cells [61]. The quercetin-induced cell cycle arrest reported above in HL-60 cells was similarly observed by Lee and colleagues in a different human leukemia cell lines namely, U937 cells. Cell cycle was arrested at G2/M phase in a time-dependent manner following treatment of the U937 cells with 20 μM quercetin and this effect was associated with significant decline in the expression of cyclin D, cyclin E, E2F1, and E2F2, and a concomitant up-regulation of cyclin B; it was however not associated with obvious changes in the concentrations of cdc2, cdk4, and cdk2 proteins. They also demonstrated a dose-dependent quercetin-induced DNA fragmentation with typical ladder appearance (a common feature of apoptosis) in the treated cells, which they were able to confirm that it was mediated by caspase-3 as evident by increased proteolytic cleavages of PLC-γ1 (a target of caspase-3). They concluded that quercetin exhibited dual mechanisms of actions namely: Cell cycle arrest at G2/M phase and induction of apoptosis through caspase activation [62]. Recent literature has now provided evidence that quercetin is a pleiotropic molecule with diverse activities that are believed to be directed towards inhibition of cancer cell growth. Spagnuolo and colleagues have demonstrated this property by first showing the ability of quercetin to improve apoptotic activity of anti-CD95 and recombinant tumour necrosis factor-alpha-related apoptosis-inducing ligand (rTRAIL) in acute lymphocytic leukemia. They then isolated B cells from peripheral blood of chronic lymphocytic leukemia patients (specifically those with high resistance to apoptotic effect of anti-CD95 and rTRAIL and as well as low sensitivity to quercetin). Treatment of these cells with quercetin showed improved sensitivity to anti-CD95 and rTRAIL with increase in number of dead cells by about 1.5 and 1.6-fold respectively when compared with treatment of the cells with quercetin alone [63].

### 2.2. Kaempherol

Bestwick *et al.*, have studied the effects of kaempferol on HL-60 cells and reported a dose dependent growth inhibition that was associated with significant disruption in the cell cycle of these cells after treatment with ≥10 μM kaempferol. These changes were obvious by 5 h after treatment; an initial treatment with 1 μM kaempferol did not show any significant change but with increase dose of ≥10 μM kaempferol, there was an increase in the proportion of cells in S-phase and decrease in G1-phase and at a dose of 50 μM there was a marked increase in G2-M phase cells. They attributed these changes to kaempferol -induced DNA damage and therefore proposed that the cytotoxic effects of kaempferol come from both apoptotic and non-apoptotic activities [64].

### 2.3. Galangin

Galangin (3,5,7-trihydroxyflavone) is a flavonoid present in honey. It has been demonstrated to have antiproliferative effect on HL-60 cell line. This effect was dose dependent and following 24 h treatment of the cells with the highest dose of galangin (100 μM), a marked decrease in the number of cells was observed. In addition, a dose of ≥10 μM galangin was associated with a progressive reduction in membrane integrity and viability as evident by increased trypan blue staining. Elevated levels of caspase 3, a common characteristic of apoptotic changes, was observed following 24 h and 72 h incubation with ≥50 and >10 μM of galangin respectively [65].

### 2.4. Apigenin

Wang and colleagues have reported the effect of apigenin (40,5,7-trihydroxyflavone) on apoptosis in HL-60 cells. A dose dependent DNA fragmentation was observed after 12 h treatment of HL-60 cells with 20, 40 and 60 μM apigenin; DNA fragmentation then became obvious between a dose of 20 and 40 μM apigenin. However, when the dose was increased to 60 μM apigenin, there were visible DNA ladders just 6 h after treatment, and subsequently an increasing DNA fragmentation was noted from 6 to 12 h treatment. They also noted a dose and time dependent increased release of cytochrome c and subsequent elevation of activities of caspase-3 and -9 proteases following treatment of the HL-60 cells with apigenin. Hence, they suggested that the induction of apoptosis by apigenin and other flavonoids may be mediated by activation of caspase-3 protease [66]. Sequel to association of heat-shock proteins-27 (Hsp27) with regulators of extrinsic and intrinsic apoptotic cell death as well as its role in increasing progression of tumour growth and resistance to anti-cancer therapy, its regulation by apigenin was investigated in THP- leukemia cells by Gonzalez-Mejia and colleagues. Apigenin produced no effect on the total expression of Hsp27; however it caused phosphorylation of Hsp27 in a unique bimodal fashion. An early p8-MAPK-regulated phosphorylation of Hsp-27 was observed on only Ser78 (S78) and Ser82 (S82) after 15 min of incubation of the THP-cells with apigenin; on the contrary, a late phosphorylation of all the three residues of Hsp27 (S15, S78, and S82) was observed following 6 h treatment with apigenin. The early phosphorylation was found to be regulated by p38 whereas the late one was regulated by both p38 and PKCδ. There was also associated increased activity of phosphor-mimic mutant Hsp27-D which is believed to increase the susceptibity of leukemia cells to apigenin-induced apoptosis by raising the number of apoptotic cells following 9 h treatment [67].

Recent study by Budhraja and colleagues demonstrated that apigenin is a pleiotropic compound by possessing multiple mechanisms of inducing apoptosis *in vitro* as well as inhibiting tumour formation *in vivo*. First they showed that it induced a caspase-dependent apoptosis in U937 cells in a dose and time-dependent manner. Specifically, apigenin caused activation of caspases-3, -7, and -9 as well as cleavage of PARP in the treated U937 cells. There was also a subsequent down-regulation of Bcl-2 and Mcl in a similar dose and time-dependent manner. Other effects observed following treatment of U937 cells with apigenin include induction of mitochondrial injury, inactivation of Akt and activation of c-jun-NH_2_-kinase (JNK). Following inoculation of U937 human leukemia xenografts in mice, apigenin was found to exhibit significant growth inhibition in the cells of experimental group compared to the control. There was a concomitant over-expression of phospho-JNK, cleavage of PARP, and down-regulation of Akt and Mcl-1 in the tested group compared with the vehicle group. Hence, this confirmed apoptotic process in apigenin-treated U937 xenografts mice. There was also a close correlation between levels of activity of phosphor-Akt, phospho-JNK, PPRP, and Mcl-1 in the tissue xenograft and the reduction in size of the U937 tumor xengrafts [68].

### 2.5. Acacetin

The antileukemic effect of acacetin has been recently studied in human T cell leukemia jurkat cells and was shown to inhibit their growth by a dose and time-dependent induction of apoptosis. There was also associated activation of caspase-3, -8, and -9 in a similar dose-dependent manner. Incubation of the jurkat cells with acacetin also enhanced the expression of FAF1, phosphor-FADD, Apaf-1 and cytochrome c. There was also a concomitant increased activity of Bax and decreased expression of Bcl-2. It was therefore concluded that the acacetin-induced apoptosis was probably regulated by Fas-mediated mechanism [69].

### 2.6. Fisetin, Myricetin and Wogonin

Fisetin and wongonin are two related flavonoids and their anticancer properties in HL-60 cells were studied extensively. Lee *et al*. have observed a dose dependent induction of apoptosis in HL-60 cells with doses of 20, 40 and 80 μM fisetin and myricetin. They demonstrated increased apoptotic bodies by microscopic observation and enhanced sub-G1 ratio following treatment of the HL-60 cells with Fisetin and wongonin and this was accompanied by activation of caspase-3 with an attendant PARP cleavage (a feature of apoptosis). They therefore suggested that a neutral Ca^2+^/Mg^2+^ dependent endonuclease could play a central role in the apoptotic activity of fisetin and wogonin [70]. Ding *et al.*, have reported that Wogonin and its structurally related natural flavones apigenin and chrysin possess the ability to sensitize human T cell leukemia virus type 1 (HTLV-1)-associated adult T cell leukemia/lymphoma (ATL) cell line to TRAIL induced apoptosis. The authors attempted to answer the question whether these flavones possess the ability to reduce the activity of c-FLIP (believed to play a vital role in resistance to TRAIL-induced apoptosis) in the tested cells by selecting two ATL cell lines, SP and MT-2, obtained from HTLV-1-infected ATL patients. They confirmed over-expression of c-FLIP by comparing its activity in SP and MT-2 ATL cells with a control (non-HTLV-1-infected*Int.* leukemic jurkat cells) and they found it to be 5–9 folds higher in the ATL cells than the control cells. Following treatment of the SP and MT-2 cells with 50 μM wogonin, apigenin, and chrysin, a down-regulation of c-FLIP mRNA expression was observed, and this was correlated with decrease in expression of c-FLIPs protein levels. Similar result was also obtained for Mcl-1 protein. The down-regulation of c-FLIP was subsequently demonstrated to correlate with a dose-dependent sensitization of TRAIL-induced apoptosis in the resistant SP and MT-2 cells following treatment with the three flavones. Furthermore, increased cleavage of caspase-8 and Bid was also observed when SP and MT-2 were treated with a combination of TRAIL and wogonin. Wogonin, apigenin, and chrysin were also found to enhance TRAIL-R2 expression in the tested cells which may further facilitate TRAIL-induced apoptotic death in those cancer cells resistant to treatment with TRAIL alone [71].

### 2.7. Caffeic Acid Phenylethyl Ester (CAPE)

In 1996, Chen and co-workers examined the antiproliferative effect of CAPE in HL-60 cells and reported a dose dependent growth inhibition in these cells. They observed that in 48 h, CAPE at doses of 0.16, 0.62, 2.5, 10 μM caused cell growth inhibition by 15%, 37%, 54%, and 74% respectively. There was also an accompanying inhibition of DNA, RNA, and protein synthesis in the HL-60 cells with doses of 10 and 40 μM CAPE capable of causing a complete inhibition of DNA and protein synthesis and RNA synthesis respectively [72]. Later on, in 2001, Yu-Jen Chen *et al*. further elaborated on the apoptosis-inducing effect of CAPE in human myeloid leukemic cell line (HL-60). Their findings indicated a dose and time dependent induction of apoptosis (evident by cytoplasmic blebbings and chromatin condensation) after treatment with CAPE. A dose of 6 μg/mL CAPE induced apoptosis in about 25% and 67% of the HL-60 cells following 6 and 72 h treatment respectively. There were also associated changes such as cleavage of caspase-3, inhibition of Bcl-2 expression, and stimulation of Bax [73]. In a more recent study conducted by Jin *et al*., it was discovered that CAPE induced mitochondria-mediated apoptosis in U937 cells. They analyzed the cytosolic cytochrome c in U937 cells that were treated with various concentrations (0, 0.01, 0.1, 1, and 5 μg/mL) of CAPE and found a significant elevation of the protein; in addition, they ruled out possibility of other mechanisms of apoptosis playing any role in the apoptotic activities observed, by examining death receptor and ER-mediated mechanisms; their result neither showed expression of Fas protein (which plays role in death-receptor mediated apoptosis) nor did it show phosphorylation of eIF2 or expression of CHOP (both important in ER-mediated apoptosis) following incubation of U937 cells with CAPE. On account of this, they concluded that the apoptosis induced by CAPE in U937 was mitochondria-mediated and not death receptors or ER mediated type [74].

### 2.8. Chrysin

Chrysin (5,7-dihydroxyflavone) is a natural flavonoid abundantly found in plants, honey and propolis. Woo *et al*. have demonstrated that treatment of U937 cells with different doses of chrysin was associated with induction of apoptosis, which was accompanied by significant accumulation of the sub-G1 phase. To delineate the contribution of caspases in chrysin-induced apoptosis, they determined the activity of caspase-3 by treating the U937 cells with 5–10 μM chrysin for 12 h; this led to a decrease in the levels of pro-caspase 3. They then limited the dose of chrysin to 5 μM and treated the cells for 12 h and then observed increased proteolytic cleavages of PLC-γ1 (a target of caspase-3) and DEVD-pNA, which is also a caspase-3 substrate. It was therefore concluded by these authors that, caspase-3 played a crucial role in Chrysin-induced apoptosis in U937 cells and that the induction of apoptosis was by deactivation of P13K/Akt signal pathway as well as inhibition of NF-κB and down-regulation of XIAP [75]. 5,7-dimethoxyflavone (DMF) which is a methylated analog of chrysin, have been reported to have similar effects with the former on YCUB leukemia cell lines. Treatment of these cells with DMF showed a dose-dependent inhibition of their growth with a concomitant reduction in cellular glutathione (GSH) levels. 20 μg/mL of DMF caused cell cycle arrest at G_o/_G_1_ phase in YCUB-2 cells in a time-dependent manner. There was also an accompanying decrease in number of YCUB-2 cells at S and G_2_/M phases in a similar fashion. Measurement of annexin V-positive YCUB-2 cells revealed DMF-induced apoptotic cell death in a time-dependent manner with significance apoptosis being achieved only after a long duration of treatment. Exposure of YCUB-2 and YCUB-5 to 5 or 20 μg/mL for 24 h revealed accumulation of ROS in YCUB-5 cells, but not in YCUB-2 cells, hence, suggesting that DMF-induced growth inhibition was not through oxidative stress-dependent pathway. Treatment of YCUB-2, YCUB-5, and YCUB-6 cells with a combination of DMF and other anticancer drugs [4-hydroperoxy-cyclophosphamide (4HO-CY), cytarabin (AraC), l-asparaginase (l-asp), or vincristine (VCR)] indicated that DMF exhibited antagonistic effects on all the tested drugs, it was therefore suggested by the authors that chrysin and its analog (DMF) should be used each as single therapeutic agents rather than in combination with other anticancer drugs [58].

Based on the available reports that Methoxyflavones (MF) possesses more chemopreventive effect than flavones, Wudtiwai and colleagues examined the role of combination of TRAIL and MF derivatives on apoptotic induction in MOLT-4 and U937 cells. They selected five different MF namely, 2′-methoxyflavone (2′-MF), 5-methoxyflavone (5-MF), DMF, 5,7,4′-trimethoxyflavone (TMF), and 3,5,7,3′,4′-pentamethoxyflavone (PMF) and demonstrated that all of them were toxic to MOLT-4 and U937 cells. However, both cells were resistant to treatment with TRAIL alone. When the cells were treated with a combination of each of the MF derivatives with TRAIL, a significant apoptotic cell death was observed in MOLT-4 cells; on the contrary, this effect was not seen in U937 cells. The combined treatment also caused an increase in percentage of MOLT-4 cells with reduced mitochondrial transmembrane potential compared to treatment with TRAIL alone. In order to determine the mechanism of how 5-MF and 2′-MF potentiate TRAIL-induced apoptosis in MOLT-4 cells, their effect on expression of TRAIL-R1 (DR4) and TRAIL-R2 (DR5) receptors was studied. It was observed that only 5-MF was capable of up-regulating the expression of both DR4 and DR5 in a time-dependent manner. Similarly, treatment with 5-MF caused a time-dependent down-regulation of anti-apoptotic proteins cFLIP and Mcl, as well as up-regulation of Bax with concomitant cleavage of Bid. The apoptosis induced by either 5-MF or 2′-MF was found to be mediated via the extrinsic pathway by increasing the activity of caspase-8. Furthermore, there was a time-dependent increased caspase-3 activity following treatment of MOLT-4 cells with combination of either 2′-MF or 5-MF with TRAIL, it was therefore concluded that the combined treatment induced apoptosis via caspase-3 acivation [76]. This synergistic effect of combination of MF and TRAIL however contradicts the above report of Goto *et al*., where combination of DMF and other anticancer drugs (4HO-CY, AraC, l-asp, or VCR) was shown to be antagonistic instead of synergistic.

### 2.9. Luteolin

Luteolin has also been reported to exhibit anti-cancer properties. The growth of HL-60 cells treated with different doses of luteolin was inhibited in a dose-dependent manner; this was then followed by cytoplasmic blebbings, cell shrinkage, chromatin condensation, appearance of apoptotic bodies, and appearance of DNA ladder pattern in the luteolin treated HL60 cells [77]. More details on the mechanism of induction of apoptosis by luteolin were examined by Cheng *et al.* They observed dose and time-dependant induction of apoptosis after treatment of human promyelocytic leukemia HL-60 cell lines with 60–100 μM luteolin. A dose of 60 μM luteolin induced a time dependant apoptotic cell death from 3.2% (3 h) to 33.6% (6 h). They also demonstrated a dose and time-dependant DNA fragmentation with visible DNA ladders, as well as disruption of mitochondrial membrane potential with consequent release of cytochrome c in to the cytosol, which in turn caused activation of caspase-3 and caspase-9 following treatment of HL-60 cells with 20–60 μM luteolin. It was therefore concluded that luteolin induced apoptosis in HL-60 cells through mitochondrial pathway [78].

### 2.10. Hesperidin

Hesperidin has been shown to exert anti-cancer effects on wild-type p53-positive human pre-B cell (NALM-6) cell line. Treatment of these cells with 10–100 μM hesperidin caused dose and time-dependant growth inhibition associated with over-expression of PPARγ. A dose of 10–50 μM hesperidin was however enough to induce apoptosis in NALM-6 cells and the apoptotic process was caspase dependant (evidenced by increased cleavage of caspase-3 and caspase-9). There was also concomitant up regulation of Bax protein expression as well as down-regulation of Bcl-2 and XIAP following treatment of the NALM-6 cells with 10–100 μM hesperidin. Interestingly, a dose-dependent up-regulation of p53 expression was observed after incubation of NALM-6 cells with hesperidine, hence, this suggests that p53 might play a vital role in hesperidin-induced cell death [79].

### 2.11. Dicaffeoylquinic Acids and Caffeoylquinic Acid Derivatives

4,5-di-Ocaffeoylquinic acid (4,5-diCQA), 3,5-di-Ocaffeoylquinic acid (3,5-diCQA), and 3,4-di-Ocaffeoylquinic acid (3,4-diCQA) are three Dicaffeoylquinic acids isolated from water extract of propolis and studied by Mishima *et al.* A dose dependent arrest of cell growth was observed following incubation of HL-60 cells with doses of 40, 80, and 120 μM each of 4,5-diCQA, 3,5-diCQA, and 3,4-diCQA. The same range of doses of these compounds caused differentiation of HL-60 cells in to granulocytes with subsequent induction of nuclear condensation and fragmentation as well as formation of apoptotic bodies (characteristics of apoptosis). The authors also examined the effects of derivatives of caffeoylquinic acids such as caffeic acid, quinic acid, monocaffeoylquinic acid (chlorogenic acid) and 3,4,5-tri-O-caffeoylquinic acid (3,4,5-triCQA) in propolis on HL-60cell growth and differentiation, they obtained the following findings: 3,4,5-triCQA arrested cell growth with accompanying apoptotic changes, both caffeic and chlorogenic acids exhibited HL-60 growth inhibition only but not apoptosis induction, quinic acid on the other hand showed neither growth inhibitory nor cellular differentiation effects in the HL-60 cells. It can therefore be presumed that the potency of quinic acid to induce differentiation of HL-60 cells in to granulocytes is dependent on the amount of caffeoyl groups attached to it [80].

### 2.12. Genkwanin

Genkwanin has been reported to be cytotoxic to human chronic myelogenous leukemia (K562) cells at an IC_50_ of 7.915 × 10^−7^ mol/mL [81].

### 2.13. Rosmarinic Acid and Derivatives

Recently conducted research by Valdes and co-workers on anti-leukemic activity of three rosemary polyphenols namely, rosmarinic acid, carnosol, and carnosic acid has shed more light on the molecular mechanism of action of phenolic compounds. They used two phenotypes of K562 cells, daunomycin (DNM)-sensitive (K562wt) and DNM-resistant (K562/R). Administration of 5 and 10 μM of rosemary polyphenols was found to cause a dose and time-dependent inhibition of growth as well as apoptosis (evident by accumulation of sub-G1 cell population) in both phenotypes. Treatment of the leukemia cells with these phenolic compounds also caused an appreciable level of chemopreventive action by increasing the activity of antioxidant and phase II detoxifying enzymes (NQO1, GST, SULT, and OSGIN 1) via activation of genes that are responsible for encoding such enzymes. Similar treatment also indicated that the rosemary polyphenols caused increased levels of reduced glutathione (GSH) and reduced levels of hypoxanthine and these effects were more pronounced in the K562/R than k562 cell line. This further signifies that the K562/R cells are more responsive to the chemopreventive and antioxidant effects of the polyphenols extract than their counterpart K562 cells [82].

### 2.14. Ellagic Acid (EA)

Ellagic acid has been demonstrated to induce dose dependent cell growth inhibition and apoptosis (evident by nuclear shrinkage, chromatin condensation, and nuclear fragmentation) in HL-60 cells. The combination of these features as well as the ability of eallagic acid to induce annexin V binding makes it a potent apoptotic inducer in HL-60 cells. Incubation of the cells with 25 μM EA for 96 h caused over-expression of cleaved caspase-3, death receptors (TRAIL receptor 1 and 2, Fas), and SMAC/Diablo. There was also associated cleavage of PARP, which further confirmed that the apoptosis induced by EA was through the caspase-dependent pathway. Another interesting finding about EA is its ability to sensitize HL-60 cells to undergo myeloid differentiation with a lower dose of All-trans retinoic acid [83].

### 2.15. Other Phenolic Compounds

Pozo-Insfran and colleagues have studied the antiproliferative and apoptotic effects of a mixture of phenolic compounds extracted from a plant called *Euterpe oleracea* Mart (acai). Prominent among these compounds included ferulic acid, *p*-hydroxybenzoic acid, gallic acid, vanillic acid, and *p*-coumaric acid. HL-60 cells treated with these compounds exhibited a dose and time-dependent growth inhibition and apoptotic activities which was preceded by activation of caspase-3 [84].

Ninomiya and co-workers made another vital contribution to the role of phenolic compounds as anti-leukemic agents by studying the structure-activity relationship of 5,7-dihydroxyflavones with regards to their inhibitory effect on HL-60 cells. Although, of all the synthetic dihydroflavones investigated in these study only apigenin, acacetin, and tricetin have been confirmed to be present in honey by our search, it is still worthwhile to review all the dihydroflavones reported in the study since they are structurally related and newer information regarding the phenolic contents of honey is still emerging. The authors synthesized ten dihydroflavones (apigenin, acacetin, Diosmetin, Chrysoeriol, 3′,4′-Dimethoxyluteolin, tricetin, 4′-methoxytricetin, tricin, apometzgerin, and 3′,4′,5′-trimethoxytricetin) with a view to investigate the effect of hydroxylation and methylation in their B ring. 48 h incubation of HL-60 cells with 25–50 μM of the flavones showed a dose dependent inhibition of proliferation of these cells by all the compounds with diosmetin, chrysoeriol, and tricetin capable of inducing about 80% inhibition. Of all the five flavones with three oxygenated functional groups (tricetin, 4′-methoxytricetin, tricin, apometzgerin, and 3′,4′,5′-trimethoxytricetin); tricetin, which was the most hydroxylated, showed a more inhibitory activity than the rest of the four flavones. This therefore indicates that the effect of these compounds on HL-60 cell was affected by the combination of hydroxy and methoxy groups in the B ring of 5,7-dihydroxyflavones. Diosmetin, chrysoeriol, and tricetin at a dose of 50 and 100 μM induced nuclear fragmentation and chromatin condensation with tricetin showing a poorer apoptotic effect. Examination of differentiation (granulocytic and monocytic) inducing ability of the flavones revealed a dose-dependent improvement in the degree of cell differentiation when HL-60 cells were treated with diosmetin and chrysoeriol. Similar treatment with tricetin and tricin demonstrated a weak ability to induce the differentiation [85]. The authors however did not establish the exact mechanism involved in the ability of these compounds to induce cell differentiation; however, they postulated that this effect could result from activation of PKC and inhibition of topoisomerase activity as reported with other compounds elsewhere.

The anti-leukemic activities of various phenolic components of honey are summarized in Table 2[58,60–62,64–70,72,74,75,77,79–82,84].

## 3. Conclusions

With a rising number of new cases of all types of cancers worldwide and in particular hematologic malignancies, novel agents that could cure or prevent cancers are still very much needed. Most anticancer agents act by induction of apoptosis, cell cycle arrest, as well as an inhibition and proliferation of cell growth. This review indicates that many of the phenolic contents of honey have been shown to have anti-leukemic activities and that they exert their effects through one of the aforementioned mechanisms. It is expected that the effects of polyphenols on cell cycle, cell growth and proliferation, as well as induction of apoptosis in leukemia cancer cells, will provide clues for the prediction of novel agents that may be useful in cancer chemoprevention or chemotherapy. However, while the combination of some polyphenols enhances the antileukemic activity of some agents, other reports indicate that such combinations are not always beneficial in terms of potentiating the action of other antileukemic agents. Moreover, the concentrations of different polyphenols in honey and the interactions between them have yet to be fully elucidated; therefore, more information is needed with regards to the possible role of honey in cancer prevention and therapy.

It is important to mention that up to the time of this review, no available report on the effects of crude honey on leukemia cells existed, be it *in vitro*, *in vivo* or by clinical trial; hence, further research is necessary to elucidate the possible chemotherapeutic and chemo preventive potentials of crude honey on leukemias by using cell lines and animals, as well as conducting both clinical trials and epidemiological studies.

## Figures and Tables

**Table 1:** Phenolic compounds found in honey.

Category	Compound
Flavonoles	Quercetin, kaempferol, Galangin, Fisetin, Myricetin
Flavanones	Pinocembrin, Naringin, Naringenin, Hesperidin Pinobanksin
Flavones	Apigenin, Acacetin, Chrysin, Luteolin Genkwanin, wogonin, tricetin
Phenolic acids	Caffeic acid, chlorogenic acid, cinnamic acid, p-coumaric acid, vanillic acid, ferulic acid, p-hydroxybenzoic acid, gallic acid, syringic acid abscisic acid *, rosmarinic acid and derivatives
Coumarins	Coumarin
Tannins	Ellagic acid

* Abscisic acid doesn’t completely fit in to phenolic acids category; it however exhibits a lot of chromatographic similarity with the phenolic acids in honey.

**Table 2:** Summary of anti-leukemic effects of phenolic constituents of honey in various cell lines.

Phenolic compound	Cell line	Mechanism of action (s)
Quercetin	HL-60	Cell cycle arrest, inhibition of growth and proliferation
	K562	Induction of apoptosis and differentiation
	U937	Induction of apoptosis cell cycle arrest
Kaempferol	HL-60	Cell cycle arrest and growth inhibition
Galangin	HL-60	Antiproliferation and induction of apoptosis
Apigenin	HL-60	Induction of apoptosis
	THP	Induction of apoptosis
	U937	Induction of apoptosis
Acacetin	Jurkat cells	Growth inhibition and induction of apoptosis
Fisetin, myricetin and wogonin	HL-60	Induction of apoptosis
CAPE	HL-60	Growth inhibition and induction of apoptosis
	U937	Induction of apoptosis
Chrysin	U937	Induction of apoptosis
	YCUB series	Growth inhibition and induction of apoptosis
Luteolin	HL-60	Growth inhibition and induction of apoptosis
Hesperidin	NALM-6	Growth inhibition and induction of apoptosis
Dicaffeoylquinic acids and caffeoylquinic acid derivatives	HL-60	Growth inhibition, induction of apoptosis and Differentiation
Genkwanin	K562	Cytotoxicity
Rosmarinic acid and derivatives	K562	Growth inhibition and induction of apoptosis
Other phenolic acids	HL-60	Growth inhibition and induction of apoptosis
